# Supplementary material for: Dating the Emergence of Human Endemic Coronaviruses
Source: Viruses. 2022 May 19;14(5):1095. doi: 10.3390/v14051095 (PMC9148137; doi:10.3390/v14051095)

# Dating the emergence of human endemic coronaviruses

## Supplemental Information

**Table S1.** List of endemic coronavirus strains.

HCoV-OC43

| Accession ID | Isolate | Collection Date |
|---|---|---|
| NC_006213 | ATCC VR-759 | 1967 |
| KY014281 | 2002-04 | 2002 |
| JN129834 | HK04-01 | 2004 |
| JN129835 | HK04-02 | 2004 |
| AY903459 | 87309 | 2004 |
| AY903460 | 19572 Belgium 2004 | 2004 |
| KY014282 | 2007-09 | 2007 |
| KY983583 | HCoV_OC43/Seattle/USA/SC2481/2015 | 2015 |
| KY983585 | HCoV_OC43/Seattle/USA/SC2854/2015 | 2015 |
| KY983588 | HCoV_OC43/Seattle/USA/SC3118/2015 | 2015 |
| KY967356 | HCoV_OC43/Seattle/USA/SC2924/2015 | 2015 |
| KY967358 | HCoV_OC43/Seattle/USA/SC2770/2015 | 2015 |
| KY967359 | HCoV_OC43/Seattle/USA/SC2730/2015 | 2015 |
| KY967360 | HCoV_OC43/Seattle/USA/SC2476/2015 | 2015 |
| KY967361 | HCoV_OC43/Seattle/USA/SC2345/2015 | 2015 |
| KY554972 | N07-1541B_433X | 2016 |
| KY554973 | N07-1689B_116X | 2016 |
| KY554974 | N08-33B_360X | 2016 |
| KY554975 | N09-382B | 2016 |
| KY674917 | N07-1609B | 2016 |
| KY674918 | N07-1647B | 2016 |
| KY674920 | N09-595B | 2016 |
| KY684759 | HCoV_OC43/Seattle/USA/SC2269/2016 | 2016 |
| KY369905 | HCoV_OC43/Seattle/USA/SC831/2016 | 2016 |
| KY369906 | HCoV_OC43/Seattle/USA/SC622/2016 | 2016 |
| KY369907 | HCoV_OC43/Seattle/USA/SC9741/2016 | 2016 |
| MN306036 | HCoV_OC43/Seattle/USA/SC0682/2019 | 2019 |
| MN306041 | HCoV_OC43/Seattle/USA/SC0810/2019 | 2019 |
| MN306042 | HCoV_OC43/Seattle/USA/SC0839/2019 | 2019 |
| MN306043 | HCoV_OC43/Seattle/USA/SC0841/2019 | 2019 |
| MN306053 | HCoV_OC43/Seattle/USA/SC9430/2018 | 2019 |
| MN310476 | HCoV_OC43/Seattle/USA/SC9428/2018 | 2019 |
| MN310478 | HCoV_OC43/Seattle/USA/SC0776/2019 | 2019 |
| KF530093 | OC43/human/USA/832-27/1983 | 1983 |
| KF530060 | OC43/human/USA/851-15/1985 | 1985 |
| KF530085 | OC43/human/USA/871-25/1987 | 1987 |
| KF530086 | OC43/human/USA/872-5/1987 | 1987 |
| KF530087 | OC43/human/USA/873-6/1987 | 1987 |
| KF530077 | OC43/human/USA/873-16/1987 | 1987 |
| KF530083 | OC43/human/USA/873-19/1987 | 1987 |
| KF530073 | OC43/human/USA/8912-37/1989 | 1989 |
| KF530066 | OC43/human/USA/901-33/1990 | 1990 |
| KF530065 | OC43/human/USA/901-41/1990 | 1990 |

| | | |
|---|---|---|
| KF530061 | OC43/human/USA/901-43/1990 | 1990 |
| KF530088 | OC43/human/USA/901-54/1990 | 1990 |
| KF530076 | OC43/human/USA/911-11/1991 | 1991 |
| KF530096 | OC43/human/USA/911-38/1991 | 1991 |
| KF530091 | OC43/human/USA/911-58/1991 | 1991 |
| KF530089 | OC43/human/USA/911-66/1991 | 1991 |
| KF530095 | OC43/human/USA/912-6/1991 | 1991 |
| KF530067 | OC43/human/USA/912-10/1991 | 1991 |
| KF530082 | OC43/human/USA/912-11/1991 | 1991 |
| KF530094 | OC43/human/USA/912-36/1991 | 1991 |
| KF530079 | OC43/human/USA/913-29/1991 | 1991 |
| KF530071 | OC43/human/USA/925-1/1992 | 1992 |
| KF530097 | OC43/human/USA/9211-43/1992 | 1992 |
| KF530074 | OC43/human/USA/9212-33/1992 | 1992 |
| KF530090 | OC43/human/USA/931-85/1993 | 1993 |
| KF530059 | OC43/human/USA/951-15/1995 | 1995 |
| KF530084 | OC43/human/USA/951-18/1995 | 1995 |
| KF530062 | OC43/human/USA/952-23/1995 | 1995 |
| KF530075 | OC43/human/USA/953-23/1995 | 1995 |
| KF530098 | OC43/human/USA/965-6/1996 | 1996 |
| KF530064 | OC43/human/USA/9612-9/1996 | 1996 |
| KF530078 | OC43/human/USA/9612-29/1996 | 1996 |
| KF530063 | OC43/human/USA/9612-48/1996 | 1996 |
| KF530099 | OC43/human/USA/971-5/1997 | 1997 |
| KF530072 | OC43/human/USA/9712-13/1997 | 1997 |
| KF530080 | OC43/human/USA/9712-31/1997 | 1997 |
| KF530069 | OC43/human/USA/982-4/1998 | 1998 |
| KF530081 | OC43/human/USA/991-5/1999 | 1999 |
| KF530070 | OC43/human/USA/991-19/1999 | 1999 |
| KF530068 | OC43/human/USA/007-11/2000 | 2000 |
| KF530092 | OC43/human/USA/008-5/2000 | 2000 |
| KF923905 | 229/2005 | 2005 |
| KF923889 | 1926/2006 | 2006 |
| KF923899 | 3582/2006 | 2006 |
| KF923900 | 3647/2006 | 2006 |
| KF923890 | 39A/2007 | 2007 |
| KF923892 | 5345/2007 | 2007 |
| KF923894 | 5352/2007 | 2007 |
| KF923907 | 5370/2007 | 2007 |
| KF923921 | 69A/2007 | 2007 |
| KF923891 | 5240/2007 | 2007 |
| KF923901 | 5472/2007 | 2007 |
| KF923908 | 5414/2007 | 2007 |
| KF923909 | 5442/2007 | 2007 |
| KF923910 | 5445/2007 | 2007 |
| KF923911 | 5479/2007 | 2007 |
| KF923912 | 5484/2007 | 2007 |
| KF923913 | 5485/2007 | 2007 |
| KF923914 | 5508/2007 | 2007 |

| | | |
|---|---|---|
| KF923915 | 5517/2007 | 2007 |
| KF923916 | 5519/2007 | 2007 |
| KF923917 | 5566/2007 | 2007 |
| KF923919 | 5595/2007 | 2007 |
| KF923920 | 5617/2007 | 2007 |
| KF923923 | 892A/2008 | 2008 |
| KF923922 | 8164/2009 | 2009 |
| KP198611 | 1783A/10 | 2010 |
| KF923886 | 1908A/2010 | 2010 |
| KF923887 | 1997A/2010 | 2010 |
| KF923918 | 10108/2010 | 2010 |
| KP198610 | 2058A/10 | 2010 |
| KF923893 | 2151A/2010 | 2010 |
| KF923895 | 10285/2010 | 2010 |
| KF923924 | 10290/2010 | 2010 |
| KF923888 | 2145A/2010 | 2010 |
| KF923925 | 10574/2010 | 2010 |
| KX344031 | LRTI_238 | 2011 |
| KU131570 | HCoV-OC43/UK/London/2011 | 2011 |
| KJ958218 | LY341 | 2011 |
| KJ958219 | LY342 | 2011 |
| KF923896 | 3074A/2012 | 2012 |
| KX538964 | MY-U002/12 | 2012 |
| KF923898 | 3184A/2012 | 2012 |
| KF923906 | 3194A/2012 | 2012 |
| KX538965 | MY-U208/12 | 2012 |
| KX538966 | MY-U236/12 | 2012 |
| KF923902 | 12689/2012 | 2012 |
| KF923903 | 12691/2012 | 2012 |
| KF923904 | 12694/2012 | 2012 |
| KX538967 | MY-U413/12 | 2012 |
| KX538968 | MY-U464/12 | 2012 |
| KX538969 | MY-U523/12 | 2012 |
| KF923897 | 3269A/2012 | 2012 |
| KX538970 | MY-U710/12 | 2012 |
| KX538971 | MY-U732/12 | 2012 |
| KX538972 | MY-U774/12 | 2012 |
| KX538973 | MY-U868/12 | 2012 |
| KX538974 | MY-U945/12 | 2012 |
| KX538975 | MY-U1024/12 | 2012 |
| KX538976 | MY-U1057/12 | 2012 |
| KX538977 | MY-U1140/12 | 2012 |
| KX538978 | MY-U1758/13 | 2013 |
| KX538979 | MY-U1975/13 | 2013 |
| MG197718 | YC-55 | 2015 |
| MG197709 | BJ-112 | 2015 |
| MG197710 | BJ-124 | 2015 |
| MG197715 | GZYF-26 | 2015 |
| MG197719 | YC-67 | 2015 |

| | | |
|---|---|---|
| MG197720 | YC-68 | 2015 |
| MG197716 | WZ-303 | 2015 |
| MG197711 | BJ-164 | 2015 |
| MG197712 | BJ-165 | 2015 |
| MG197721 | YC-72 | 2015 |
| MG197717 | WZ-522 | 2015 |
| MG197714 | CC-23 | 2015 |
| MG197713 | BJ-221 | 2015 |
| MG197722 | YC-207 | 2015 |
| MG977446 | TNP F1791_2 | 2016 |
| MF374983 | HCoV-OC43/USA/TCNP_0070/2016 | 2016 |
| MF314143 | HCoV-OC43/USA/ACRI_0052/2016 | 2016 |
| MG197723 | HZ-459 | 2016 |
| MG977451 | TNP 12636 | 2016 |
| MG977452 | TNP 12643 | 2016 |
| MH121121 | HCoV-OC43/USA/ACRI_0213/2016 | 2016 |
| MG977447 | TNP F1832_2 | 2016 |
| MG977448 | TNP F1833_2 | 2016 |
| MG977449 | TNP F1834_2 | 2016 |
| MG977450 | TNP F1835_2 | 2016 |
| MG977445 | TNP F1790_2 | 2016 |
| MF374984 | HCoV-OC43/USA/TCNP_00204/2017 | 2017 |
| MG977444 | TNP F1778_2 | 2017 |
| MF374985 | HCoV-OC43/USA/TCNP_00212/2017 | 2017 |
| MN026165 | OC43_KLF_02_2017 | 2017 |
| MN026164 | OC43_KLF_01_2018 | 2018 |
| NC_003045 | Bovine coronavirus (BCoV-ENT) | 1997 |

HCoV-229E

| Accession ID | Isolate | Collection Date |
|---|---|---|
| NC_002645 | Human coronavirus 229E | 1962 |
| JX503061 | J0304 | 2009 |
| JX503060 | 349 | 2010 |
| KY983587 | HCoV_229E/Seattle/USA/SC3112/2015 | 2015 |
| KY967357 | HCoV_229E/Seattle/USA/SC2872/2015 | 2015 |
| KU291448 | HCoV-229E/BN1/GER/2015 | 2015 |
| KY621348 | HCoV_229E/Seattle/USA/SC379/2016 | 2016 |
| KY674914 | HCoV_229E/Seattle/USA/SC399/2016 | 2016 |
| KY674919 | N08-434B | 2016 |
| KY684760 | HCoV_229E/Seattle/USA/SC2282/2017 | 2016 |
| KY369908 | HCoV_229E/Seattle/USA/SC579/2016 | 2016 |
| KY369909 | HCoV_229E/Seattle/USA/SC677/2016 | 2016 |
| KY369910 | HCoV_229E/Seattle/USA/SC1143/2016 | 2016 |
| KY369911 | HCoV_229E/Seattle/USA/SC1212/2016 | 2016 |
| KY369912 | HCoV_229E/Seattle/USA/SC9731/2016 | 2016 |
| KY369913 | HCoV_229E/Seattle/USA/SC1073/2016 | 2016 |

| | | |
|---|---|---|
| KY369914 | HCoV_229E/Seattle/USA/SC9773/2016 | 2016 |
| MT438696 | HCoV-229E/USA/UNM_0186/2016 | 2016 |
| MF542265 | 229E/Haiti-1/2016 | 2016 |
| MN369046 | HCoV_229E/Seattle/USA/SC9724/2018 | 2018 |
| MN306046 | HCoV_229E/Seattle/USA/SC0865/2019 | 2019 |
| KF514429 | 229E/human/USA/892-11/1989 | 1989 |
| KF514432 | 229E/human/USA/932-72/1993 | 1993 |
| KF514433 | 229E/human/USA/933-40/1993 | 1993 |
| KF514430 | 229E/human/USA/933-50/1993 | 1993 |
| KF514431 | 229E/human/USA/953-49/1995 | 1995 |
| MT438700 | HCoV-229E/USA/UAMS_DID_0108/2017 | 2017 |
| MT438697 | HCoV-229E/USA/ACRI_0244/2017 | 2017 |
| MT438698 | HCoV-229E/USA/ACRI_0256/2017 | 2017 |
| MT438699 | HCoV-229E/USA/ACRI_0246/2017 | 2017 |
| MW039392 | HCoV 229E/human/ITA/TE5146/2020 | 2020 |
| NC_028752 | Camel alphacoronavirus (camel/Riyadh/Ry141/2015) | 2015 |

HCoV-HKU1

| Accession ID | Isolate | Collection Date |
|---|---|---|
| NC_006577 | HKU1 | 2004 |
| DQ415896 | N19 | 2004 |
| DQ415897 | N20 | 2004 |
| DQ415898 | N21 | 2004 |
| DQ415899 | N22 | 2005 |
| DQ415900 | N23 | 2005 |
| DQ415902 | N25 | 2005 |
| DQ415903 | N3 | 2003 |
| DQ415904 | N6 | 2004 |
| DQ415905 | N7 | 2004 |
| DQ415906 | N9 | 2004 |
| DQ415907 | N10 | 2004 |
| DQ415908 | N11 | 2004 |
| DQ415909 | N13 | 2004 |
| DQ415910 | N14 | 2004 |
| DQ415911 | N15 | 2004 |
| DQ415912 | N16 | 2004 |
| DQ415913 | N17 | 2004 |
| HM034837 | Caen1 | 2005 |
| KF430201 | HKU1/human/USA/HKU1-18/2010 | 2010 |
| KF430202 | HKU1/human/USA/HKU1-7/2010 | 2010 |
| KF686340 | HKU1/human/USA/HKU1-5/2009 | 2009 |
| KF686341 | HKU1/human/USA/HKU1-10/2010 | 2010 |
| KF686342 | HKU1/human/USA/HKU1-11/2009 | 2009 |
| KF686343 | HKU1/human/USA/HKU1-13/2010 | 2010 |
| KF686344 | HKU1/human/USA/HKU1-15/2009 | 2009 |
| KF686345 | HKU1/human/USA/HKU1-20/2010 | 2010 |
| KF850450 | HKU1 1102 | 2005 |

| | | |
|---|---|---|
| KT779555 | BJ01-p3 | 2009 |
| KT779556 | BJ01-p9 | 2009 |
| KY674943 | N09-1605B | 2016 |
| KY983584 | SC2628 | 2015 |
| MH940245 | SI17244 | 2017 |
| MK167038 | SC2521 | 2017 |
| NC_012936 | Murine coronavirus (Rat coronavirus Parker) | 1970 |

HCoV-NL63

| Accession ID | Isolate | Collection Date |
|---|---|---|
| NC_005831 | Amsterdam I | 2003 |
| KF530110 | NL63/human/USA/838-9/1983 | 1983 |
| KF530106 | NL63/human/USA/8712-17/1987 | 1987 |
| KF530114 | NL63/human/USA/891-4/1989 | 1989 |
| KF530108 | NL63/human/USA/891-6/1989 | 1989 |
| KF530111 | NL63/human/USA/901-24/1990 | 1990 |
| KF530109 | NL63/human/USA/903-28/1990 | 1990 |
| KF530104 | NL63/human/USA/904-20/1990 | 1990 |
| KF530113 | NL63/human/USA/905-25/1990 | 1990 |
| KF530107 | NL63/human/USA/911-56/1991 | 1991 |
| KF530105 | NL63/human/USA/012-31/2001 | 2001 |
| KF530112 | NL63/human/USA/0111-25/2001 | 2001 |
| JX504050 | NL63/RPTEC/2004 | 2004 |
| JQ765568 | NL63/DEN/2005/193 | 2005 |
| JQ765569 | NL63/DEN/2005/232 | 2005 |
| JQ765570 | NL63/DEN/2005/235 | 2005 |
| JQ765571 | NL63/DEN/2005/271 | 2005 |
| JQ900258 | NL63/DEN/2005/291 | 2005 |
| JQ765572 | NL63/DEN/2005/347 | 2005 |
| JQ900259 | NL63/DEN/2005/449 | 2005 |
| JQ765573 | NL63/DEN/2005/1062 | 2005 |
| JQ900260 | NL63/DEN/2005/1120 | 2005 |
| JQ765574 | NL63/DEN/2005/1862 | 2005 |
| JQ765575 | NL63/DEN/2005/1876 | 2005 |
| JQ765566 | NL63/DEN/2008/16 | 2008 |
| JX104161 | CBJ 037 | 2008 |
| JX524171 | CBJ123 | 2009 |
| JQ765565 | NL63/DEN/2009/15 | 2009 |
| JQ900257 | NL63/DEN/2009/31 | 2009 |
| JQ900255 | NL63/DEN/2009/6 | 2009 |
| JQ765564 | NL63/DEN/2009/14 | 2009 |
| JQ900256 | NL63/DEN/2009/22 | 2009 |
| JQ765567 | NL63/DEN/2009/20 | 2009 |
| JQ765563 | NL63/DEN/2009/9 | 2009 |
| MG428702 | Kilifi_HH_3807_11-May-2010 | 2010 |
| MG428699 | Kilifi_HH_5709_19-May-2010 | 2010 |

| | | |
|---|---|---|
| MG428704 | Kilifi_HH_5402_20-May-2010 | 2010 |
| MG428707 | Kilifi_HH_5401_20-May-2010 | 2010 |
| MG428705 | Kilifi_HH_0522_21-May-2010 | 2010 |
| MG428706 | Kilifi_HH_3808_24-May-2010 | 2010 |
| MG428700 | Kilifi_HH_1602_01-Jun-2010 | 2010 |
| MG428703 | Kilifi_HH_0511_01-Jun-2010 | 2010 |
| MG428701 | Kilifi_HH_0512_04-Jun-2010 | 2010 |
| MG772808 | CN0601/14 | 2014 |
| KY983586 | HCoV_NL63/Seattle/USA/SC2940/2015 | 2015 |
| KY829118 | N07-262B | 2015 |
| KT381875 | NL63/UF-1/2015 | 2015 |
| KT266906 | HCoV NL63/Haiti-1/2015 | 2015 |
| KX179500 | NL63/UF-2/2015 | 2015 |
| KU521535 | NL63/UF-2/2015 | 2015 |
| KY554967 | N06-1144B | 2016 |
| KY554968 | N07-185B | 2016 |
| KY554969 | N07-196B | 2016 |
| KY554970 | N07-324B_182X | 2016 |
| KY554971 | N07-468B_176X | 2016 |
| KY674915 | N07-6B | 2016 |
| KY674916 | N07-64B | 2016 |
| MN306018 | HCoV_NL63/Seattle/USA/SC0179/2018 | 2018 |
| MN026166 | NL63_KLF_01_2018 | 2018 |
| MK334044 | ChinaGD03 | 2018 |
| MK334047 | ChinaGD04 | 2018 |
| MK334045 | ChinaGD05 | 2018 |
| MK334043 | ChinaGD02 | 2018 |
| MK334046 | ChinaGD01 | 2018 |
| MN306040 | HCoV_NL63/Seattle/USA/SC0768/2019 | 2019 |
| DQ445911 | Amsterdam 057 | 2002 |
| DQ445912 | Amsterdam 496 | 2003 |
| AY518894 | Amsterdam 496 | 1988 |
| NC_032107 | NL63-related bat coronavirus (BtKYNL63-9a) | 2010 |

## Table S2. Model comparison using BEAST (v.1.10.4) path sampling.

| | | | | | | | | |
|---|---|---|---|---|---|---|---|---|
| **HCoV-OC43** | | | | | | | | |
| | | | **Strict Clock** | | | **Uncorrelated Relaxed Clock** | | |
| **Model** | | | **Constant Size** | **Exponential Growth** | **Bayesian Skyline** | **Constant Size** | **Exponential Growth** | **Bayesian Skyline** |
| | | **Likelihood** | -28557.62 | -28558.62 | -28548.08 | -28505.56 | -28507.31 | -28503.15 |
| **Strict Clock** | **Constant Size** | -28557.62 | 0 | 1 | -9.54 | -52.06 | -50.31 | -54.47 |
| | **Exponential Growth** | -28558.62 | -1 | 0 | -10.54 | -53.06 | -51.31 | -55.47 |
| | **Bayesian Skyline** | -28548.08 | 9.54 | 10.54 | 0 | -42.52 | -40.77 | -44.93 |
| **Uncorrelated Relaxed Clock** | **Constant Size** | -28505.56 | 52.06 | 53.06 | 42.52 | 0 | 1.75 | -2.41 |
| | **Exponential Growth** | -28507.31 | 50.31 | 51.31 | 40.77 | -1.75 | 0 | -4.16 |
| | **Bayesian Skyline** | -28503.15 | 54.47 | 55.47 | 44.93 | 2.41 | 4.16 | 0 |
| **HCoV-229E** | | | | | | | | |
| | | | **Strict Clock** | | | **Uncorrelated Relaxed Clock** | | |
| **Model** | | | **Constant Size** | **Exponential Growth** | **Bayesian Skyline** | **Constant Size** | **Exponential Growth** | **Bayesian Skyline** |
| | | **Likelihood** | -33495.25 | -33494.30 | -33492.58 | -33501.80 | -33502.00 | -33500.88 |
| **Strict** | **Constant Size** | -33495.25 | 0 | -0.95 | -2.67 | 6.55 | 6.75 | 5.63 |

| | | | | | | | | |
|---|---|---|---|---|---|---|---|---|
| Clock | Exponential Growth | -33494.30 | 0.95 | 0 | -1.72 | 7.5 | 7.7 | 6.58 |
| | Bayesian Skyline | -33492.58 | 2.67 | 1.72 | 0 | 9.22 | 9.42 | 8.3 |
| Uncorrelated Relaxed Clock | Constant Size | -33501.80 | -6.55 | -7.5 | -9.22 | 0 | 0.2 | -0.92 |
| | Exponential Growth | -33502.00 | -6.75 | -7.7 | -9.42 | -0.2 | 0 | -1.12 |
| | Bayesian Skyline | -33500.88 | -5.63 | -6.58 | -8.3 | 0.92 | 1.12 | 0 |
| **HCoV-NL63** | | | | | | | | |
| | | | **Strict Clock** | | | **Uncorrelated Relaxed Clock** | | |
| **Model** | | | **Constant Size** | **Exponential Growth** | **Bayesian Skyline** | **Constant Size** | **Exponential Growth** | **Bayesian Skyline** |
| | | **Likelihood** | -4363.63 | -4364.17 | -4366.40 | -4365.32 | -4366.19 | -4369.26 |
| Strict Clock | Constant Size | -4363.63 | 0 | 0.54 | 2.77 | 1.69 | 2.56 | 5.63 |
| | Exponential Growth | -4364.17 | -0.54 | 0 | 2.23 | 1.15 | 1.99 | 5.09 |
| | Bayesian Skyline | -4366.40 | -2.77 | -2.23 | 0 | -1.08 | -0.21 | 2.86 |
| Uncorrelated Relaxed Clock | Constant Size | -4365.32 | -1.69 | -1.15 | 1.08 | 0 | 0.87 | 3.94 |
| | Exponential Growth | -4366.19 | -2.56 | -1.99 | 0.21 | -0.87 | 0 | 3.07 |
| | Bayesian Skyline | -4369.26 | -5.63 | -5.09 | -2.86 | -3.94 | -3.07 | 0 |

Note: A Bayes Factor >3 indicates positive support for the row model. A strong support is usually considered when BF>20.

**Figure S1.** HCoV-NL63 and HCoV-HKU1 timescaled phylogenetic trees. Maximum clade credibility trees estimated for the non-recombinant region of HCoV-NL63 (a) and HCoV-HKU1 (b). Branch lengths represent the evolutionary time measured by the grids corresponding to the timescale shown at the tree base (in years). For internal nodes 95% HPD bars are shown and black dots indicate a posterior probability >0.80 for that node.

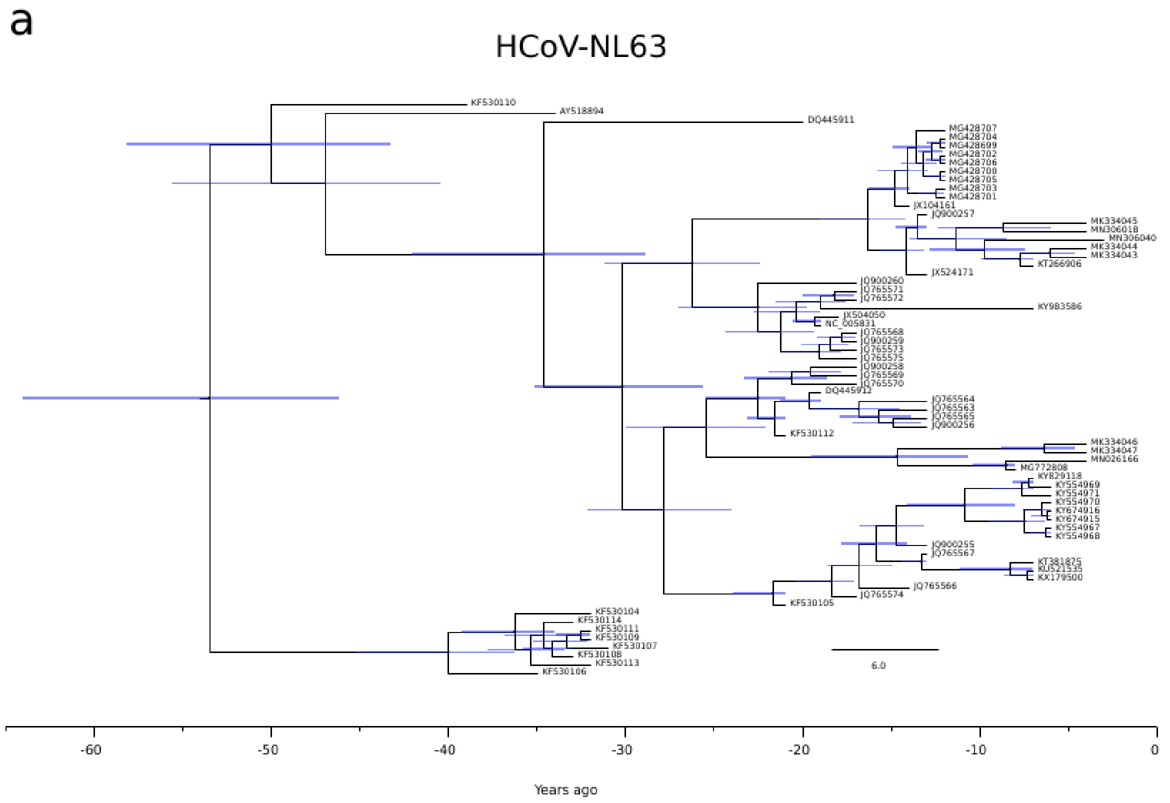


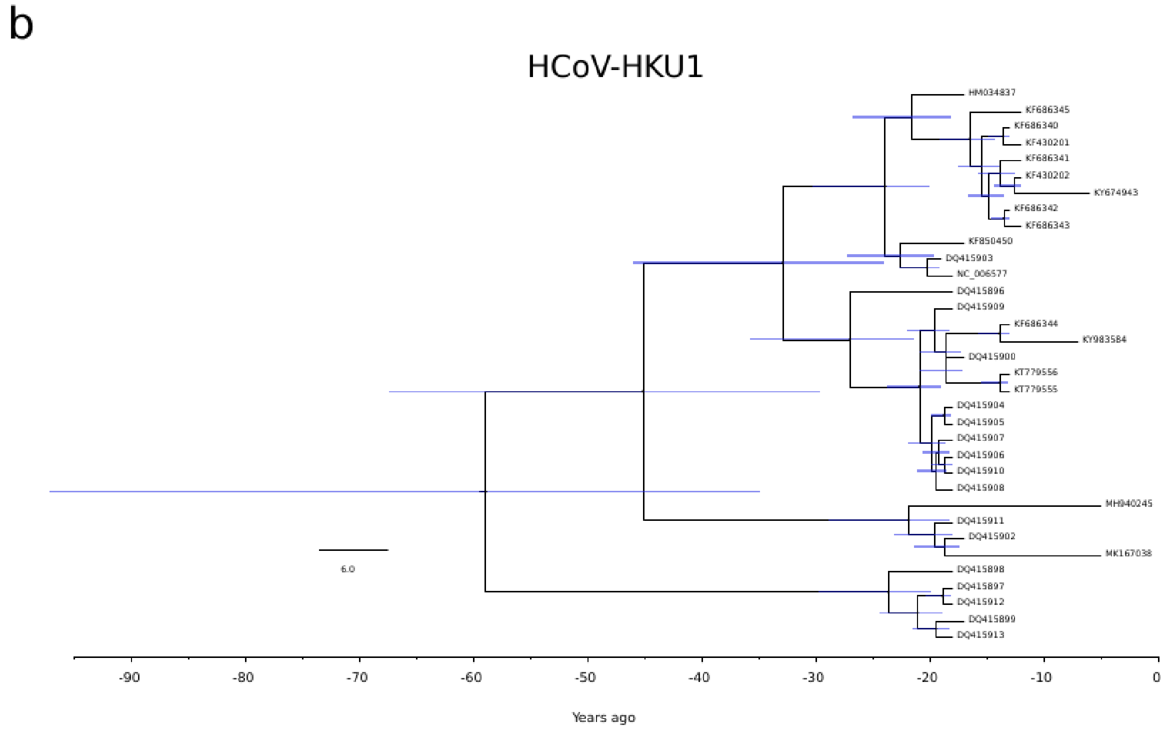

Supplement: Supplementary file 1 [file viruses-14-01095-s001.zip › viruses-1723669-supplementary.pdf]
